# Characterization of the complete chloroplast genome of *Lonicera macranthoides*

**DOI:** 10.1080/23802359.2018.1507643

**Published:** 2018-09-28

**Authors:** Huan Hu, Jianguo Liu, Jiaxing An, Miao Wang, Qian Wang

**Affiliations:** a Special Key Laboratory of Microbial Resources and Drug Development from Higher Education Institution of Guizhou Province, Research Center for Medicine and Biology, Zunyi Medical University, Zunyi, China;; b Department of Gastroenterology, Affiliated Hospital, Zunyi Medical University, Zunyi, China

**Keywords:** Chloroplast genome, *Lonicera macranthoides*, Caprifoliaceae, Phylogenetic analysis

## Abstract

*Lonicera macranthoides* is an important Chinese traditional medicine plant which is endemic in southwest China. The complete chloroplast genome from *L. macranthoides* is determined in this study. The genome size was 154,897 bp, containing a large single copy (LSC) region of 88,692 bp and a small single copy (SSC) region of 18,623 bp, which were separated by a pair of 23,786 bp inverted repeat (IR) regions. The plastome contained 130 genes, including 81 protein-coding genes (77 PCG species), eight ribosomal RNA genes (4 rRNA species), 39 tRNA genes (29 tRNA species) and two pseudognes. The most of gene species occurred as a single copy, while 13 gene species occurred in double copies, including all rRNA species, six tRNA species and three PCG species, and two tRNA genes occur in treble copies. Finally, phylogenetic analysis demonstrated that *L. macranthoides* is closely related to *L. japonica*, and genus *Lonicera* shows a closer relationship with *Triostrum* for the current data.

*Lonicera macranthoides* Hand. Mazz. is one legal resource of Lonicerae flos affirmed by the newest edition China Pharmacopeia (Committee for the Pharmacopoeia of PR China 2015). For hundreds of years, flowers of *L. macranthoides* have been used as traditional medicine for treatment of anemopyretic cold and heat-clearing (Zhao et al. 2002; Zhang et al. 2008). Good knowledge of genetics information about *L. macranthoides* can lay a good foundation for evolutionary, population genomic studies for *Lonicera*. In this study, we assembled and characterized the complete chloroplast genome sequence of *L. macranthoides* based on the Illumina pair-end sequencing data.

Fresh leaves of *L. macranthoides* were sampled from Honeysuckle plantation base of Suiyang (Zunyi, Guizhou, China; 107°8′43″ E, 27°58′38″ N). Total genomic DNA was extracted by modified CTAB method (Doyle and Doyle 1987). The whole-genome sequencing was conducted with 150 bp pair-end reads on the Illumina Hiseq Platform. About eight million high quality clean reads were obtained. Chloroplast related reads were kept by mapping to chloroplast genome of *Lonicera japonica* (KJ170923) using BWA (Li and Durbin 2009) and SAMtools (Li et al. 2009). Then, these reads were assembled into complete chloroplast genomes using Velvet (Zerbino and Birney 2008). Annotation was performed with Plann (Huang and Cronk 2015), and manually corrected with Geneious v.8.0.5 (Kearse et al. 2012) and Sequin v.15.10. The complete chloroplast genome sequence together with gene annotations, reported here for the first time, was submitted to GenBank under the accession number of MH579750 for *L. macranthoides*.

The plastome of *L. macranthoides* was highly conserved in gene order and content compared with *L. japonica* (He et al. 2017). The complete cp genome is a double stranded, circular DNA 154,897 bp in length, which contains two IR regions of 23,791 bp each separated by a LSC and a SSC region of 88,692 and 18,623 bp, respectively. The plastid genome contained 130 genes, including 81 protein-coding genes (77 PCG species), 39 tRNA genes (29 tRNA species), eight ribosomal RNA genes (four rRNA species) and two pseudogenes. The most of gene species occurred as a single copy, while 13 gene species occurred in double copies, including all rRNA species, six tRNA species and three PCG species, and two tRNA genes occurred in treble copies. The overall GC content of cp DNA was 38.51%, and 43.42% GC contents of IR regions were slightly higher than of LSC and SSC regions (36.93% and 33.52%, respectively).

A neighbor-joining tree with 1000 bootstrap replicates was performed using MEGA v7.0 (Kumar et al. 2016) from alignments created by the MAFFT (Katoh and Standley 2013). We had chosen *Sinadoxa corydalifolia* (Wang et al. 2016) as outgroup, and nine published plastomes from Caprifoliaceae as ingroups (Fan et al. 2018; Jung et al. 2018). The phylogenetic analysis indicates that *L. macranthoides* clustered together with *L. japonica*. And *Lonicera* showed a closer relationship with *Triostrum* for the current data (Figure 1). This complete chloroplast genome of *L. macranthoides* can be subsequently used for population, phylogenetic and chloroplast genetic engineering studies of the generalized honeysuckle species and *Lonicera*.

**Figure 1.**
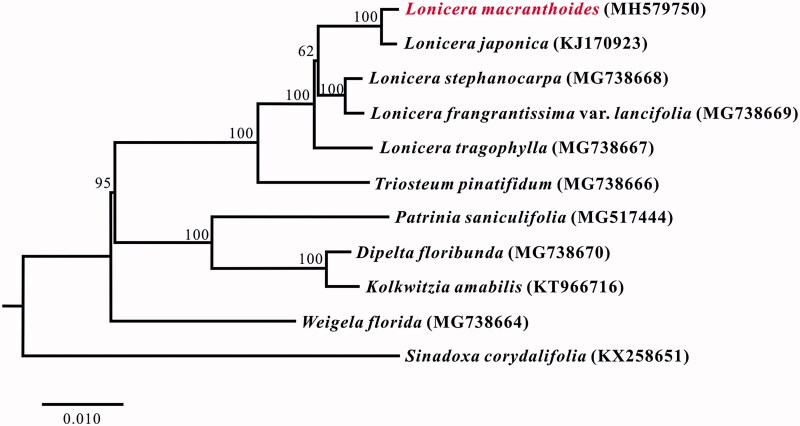
Neighbor-joining (NJ) tree based on the whole chloroplast genome sequences of 12 taxa including Lonicera macranthoides and one outgroup taxon. The whole chloroplast genome sequences were aligned using MAFFT online version (https://mafft.cbrc.jp/alignment/server/) and subjected to generating NJ phylogenetic tree by MEGA v7.0 (Kumar et al. 2016). The bootstrap support values (>50%) from 1,000 replicates are indicated in the nodes. Chloroplast genome sequences used for this tree are: *Lonicera macranthoides*, MH579750; *Lonicera japonica*, KJ170923; *Lonicera stephanocarpa*, MG738668; *Lonicera frangrantissima var. lancifolia*, MG738669; *Lonicera tragophylla*, MG738667; *Triosteum pinatifidum*, MG738666; *Patrinia saniculifolia*, MG517444; *Dipelta floribunda*, MG738670; *Kolkwitzia amabilis*, KT966716; *Weigela florida*, MG738664; *Sinadoxa corydalifolia*, KX258651 (outgroup).
